# Unraveling transcriptional control and *cis*-regulatory codes using the software suite GeneACT

**DOI:** 10.1186/gb-2006-7-10-r97

**Published:** 2006-10-25

**Authors:** Tom Hiu Cheung, Yin Lam Kwan, Micah Hamady, Xuedong Liu

**Affiliations:** 1 Department of Chemistry and Biochemistry, University of Colorado, 215 UCB, Boulder, Colorado 80309, USA; 2 Department of Computer Science, University of Colorado, 430 UCB, Boulder, Colorado 80309, USA

## Abstract

Deciphering gene regulatory networks requires the systematic identification of functional *cis*-acting regulatory elements. We present a suite of web-based bioinformatics tools, called GeneACT , that can rapidly detect evolutionarily conserved transcription factor binding sites or microRNA target sites that are either unique or over-represented in differentially expressed genes from DNA microarray data. GeneACT provides graphic visualization and extraction of common regulatory sequence elements in the promoters and 3'-untranslated regions that are conserved across multiple mammalian species.

## Abstract 2

GENEACT, a new software suite for the detection of evolutionarily conserved transcription factor binding sites or microRNAs from differentially expressed genes from DNA microarray data, is described.

## Rationale

Cell type and tissue specific gene expression patterns are primarily governed by the *cis*-regulatory sequence elements embedded in the noncoding regions of the genome. These *cis*-regulatory elements are often recognized in a sequence-specific manner by regulatory proteins or nucleic acids, which regulate the expression of the corresponding gene. In particular, activation and repression of gene transcription typically involves the binding of transcription factors to their cognate binding sites. The levels of mRNA transcript can also be modulated by microRNAs (miRNA), which tend to bind specific sequences in the 3'-untranslated region (UTR) of the transcript. Identification and characterization of *cis*-regulatory sequence elements that control gene expression are crucial to our understanding of the molecular basis of cell proliferation and differentiation.

Until recently, identification of *cis*-regulatory sequences was conducted experimentally on an individual gene basis, using time-consuming procedures such as promoter cloning, chromatin immunoprecipitation (ChIP) assays, and reporter gene assays using truncated and/or mutated DNA sequences. Given that hundreds of transcription factors regulate the expression of thousands of genes in the human genome, more high-throughput procedures are desired. The sequencing of several genomes, DNA microarray assays, and the rise of bioinformatics represent major steps forward in this regard.

Sequencing of the human, mouse, and rat genomes has made it possible to perform genome-wide analyses of regulatory sequence motifs across these species. Such a comparative genomics analysis is powerful because functional transcription factor binding sites are likely to be under stronger evolutionary constraints than random DNA sequences. Therefore, reliable and effective identification of regulatory elements could be achieved using interspecies sequence alignments of orthologous genes [1,2]. Indeed, cross-species conservation has been employed to predict conserved transcription factor binding sites and to annotate promoters in mammals [3-9]. In these cases, the comparative genomics information improved the accuracy of predicting biologically relevant transcription factor binding sites.

DNA microarray technology is used to profile relative mRNA transcript levels between samples exposed to different experimental conditions. DNA microarrays represent a high-throughput, genome-wide experimental platform that enables analyses of differential gene expression. Differences in transcript levels could be caused by several mechanisms, most notably the differential activities of transcription factors and miRNA. The interpretation of DNA microarray results requires deciphering which transcription factors and/or miRNA are likely to mediate the observed changes in transcript levels. We expect that co-expressed genes may share similar *cis*-acting regulatory elements, which suggests that such elements are likely to be over-represented in co-regulated genes more than would be expected by random chance. Flanking sequences for each gene are known from sequencing efforts, and many of the sequences to which individual transcription factors tend to bind have been determined experimentally and catalogued in databases such as the Transcription Factor Database (TFD) [10] and TRANSFAC [11]; therefore, the systematic, high-throughput prediction of specific *cis*-regulatory mechanisms important in a given biologic context is now possible. Indeed, a number of computational programs have been developed to reveal transcription factor binding sites that are statistically over-represented in co-regulated genes [12-15].

Several deficiencies exist in currently available software for predicting *cis*-regulatory elements. Most importantly, there is no program currently available that incorporates search tools for both transcription factor and miRNA binding sites. Recent studies with miRNA suggest that differential miRNA expression could be responsible for differential mRNA expression observed by DNA microarray data [16,17]. Therefore, it is imperative to investigate both transcription factor binding sites and miRNA binding sites in order to gain a more comprehensive understanding of the molecular basis of differential gene expression patterns. Second, an integrated web-based *cis*-acting element browser for rapid identification of over-represented potential transcription factor binding sites and putative miRNA target sites has yet to be developed. The lack of an easy-to-navigate graphical web interface has hindered verification of computational predictions by experimental biologists who may be less comfortable with less accessible interfaces.

In this report we describe a suite of web-based, open source bioinformatics software tools (GeneACT) that graphically display transcription factor binding sites and microRNA target sites in the regulatory regions of human, mouse, and rat genomes. In addition, we present a unique method to identify quickly transcription factor binding sites or miRNA target elements that are over-represented in differentially expressed genes based on DNA microarray data. Thus, GeneACT enables the identification of putative *cis*-acting elements that are evolutionarily conserved across species for a specified set of genes, which can be used to unravel transcriptional regulatory networks that are likely to be involved in differential gene expression.

## Development of GeneACT

**Figure 1:**
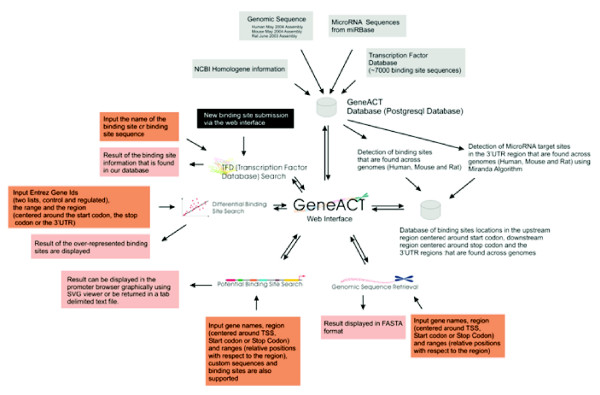
Overview of the GeneACT architecture and method.

GeneACT, an overview of which is given in Figure 1, is a suite of web-based bioinformatics tools including four useful search interfaces: differential binding site search (DBSS), potential binding site search (PBSS), genomic sequence retrieval, and TFD search. All tools are designed to characterize the regulatory regions of a specified set of genes employing the technique of comparative genomics. Genomic sequence data from human (May 2004 release), mouse (May 2004 release), and rat (June 2003 release) were downloaded from the NCBI (National Center for Biotechnology Information) ftp site [18]. TFD [19] and ortholog information (National Center for Biotechnology Information [NCBI] HomoloGene build 37.2) [20] were also downloaded from the NCBI ftp sites and employed as described below.

Detailed documentation of each of the tools in GeneACT can be found on the GeneACT website [21]. GeneACT is mainly written using Java and makes use of Tomcat as the web server. The web front end communicates with the back end via Java server page. Genomic and pre-processed data are stored in a postgreSQL database. Tutorials for GeneACT can be found on the website [21].

## Differential binding site search

**Figure 2:**
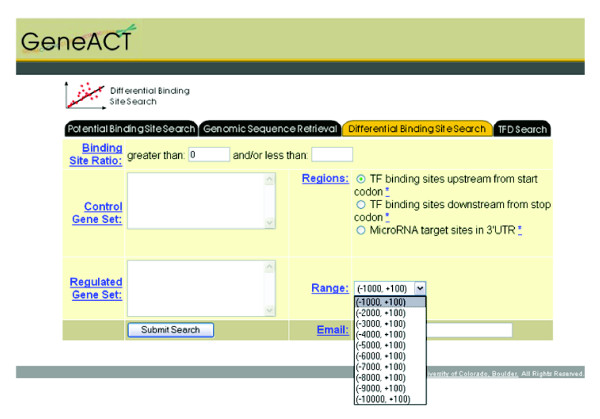
Web interface of the differential binding site search. Gene IDs from control gene set (unchanged in DNA microarray data) and regulated gene set (upregulated or downregulated from microarray data) are pasted into respective windows. The threshold of binding site ratio is defined by the user. The user can specify a range of interest with three choices of regions (upstream from the transcription start site, upstream from the start codon, or downstream from the stop codon). TF, transcription factor.

Pre-processing of sequence data underlying the GeneACT tools was carried out as follows. DBSS, the interface of which is shown in Figure 2, offers a choice of three searchable regions. The first region is denoted 'upstream of start codon', and to facilitate this search we stored the occupancies of all the binding sites in our regulatory sequence database (approximately 7000 known binding sites) in each gene found in a HomoloGene group that spans all three species up to 10,000 base pairs (bp) upstream from the start codon. We define a conserved binding site as one that is found in each of the three species within the search region, and only those binding sites that are conserved are stored for DBSS. Although promoters are frequently found near the 5'-UTRs, it is often the case that regulatory regions can be thousands of base pairs away from the transcriptional start site (for example, distal enhancers) [22-24]. As a result, we extended our search region up to 10,000 bp away from the start codon in order to cover the region of the 5'-UTR and regions that might contain these distal enhancers.

The second option for searchable region is 'downstream of stop codon'. Similar pre-processing was done for the downstream region from -2000 to +100 (2000 bp downstream of the transcript end) with respect to the stop codon. All incidences of transcription factor binding sites spanning all three species were also stored for this region. Finally, we offer a search option dedicated to detecting the occurrences of miRNA binding sites. In this case, the 3'-UTRs, defined as the region between the stop codon and the polyA signal, were extracted from the genome assemblies, and we employed miRanda [25], which is an algorithm for finding miRNA targets sites in 3'-UTRs [26]. This algorithm is based on a modified version of the Smith-Waterman algorithm [27]. Instead of building an alignment based on matching nucleotides, its score is based on the complementarity of nucleotides; this also allows G = U 'wobble' pairs, which are important for RNA:RNA duplex formation [28]. In addition, free energy is also calculated to estimate the energetics of the RNA:RNA complexes using the Vienna library. This feature makes the algorithm a preferred choice in searching for miRNA recognition sites because miRNAs form imperfect base pairs with the target mRNA [26]. To provide more stringent search results, we deposited into our database only the mature miRNA sequences from the miRBase database [29] that are absolutely conserved in all three species.

3'-UTRs from all three mammalian genomes are extracted and individually searched for potential miRNA target sites. Using the approach developed by Enright and coworkers [26], we pre-processed all three genomes individually for potential miRNA target sites. In order to count as a potential miRNA target site, we required the miRNA target sites to be found in each of the three genomes. Furthermore, it is speculated that multiple occurrences of the same miRNA target sequence in the 3'-UTR of a given mRNA increases the probability of it being regulated by that miRNA. Therefore, we introduced customizable searches by filtering the target sites into three categories based on the number of conserved matches found. In the first case, at least one conserved match must be present in the 3'-UTR of the target mRNA. For the second and the third cases, at least two or three conserved matches of the same miRNA must be present in the same target mRNA 3'-UTR, respectively. To qualify as a potential target site, the miRNA target site must be conserved across all three genomes. Users can access the database via the GeneACT web interface [30].

## Potential binding site search

**Figure 3:**
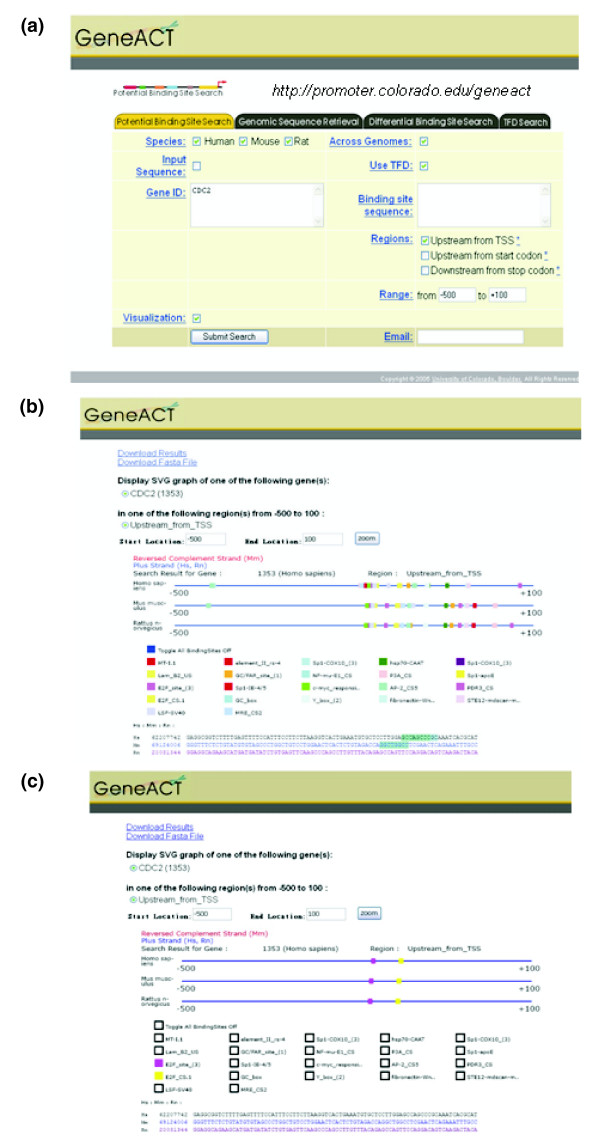
Web interface of the potential binding site search. **(a) **Web interface of potential binding site search. Gene IDs can be input in the form of either gene names (synonyms supported) or NCBI Entrez gene ID. There are currently three species to choose from (human, mouse, and rat) and it is optional to display whether the binding site sequence goes across genomes or to display all binding sites regardless of conservation across species. The user can specify a range of interest with three choices of regions (upstream from the transcription start site, upstream from the start codon, or downstream from the stop codon). Other than binding sites in the Transcription Factor Database (TFD), the user can input binding site sequences using standard IUB/IUPAC nucleic acid codes. For output option, the user can choose the visualization option for the promoter browser or a text file output. **(b) **Visualization of the *CDC2 *upstream region using GeneACT promoter browser. *CDC2 *upstream region (-500 to +100 base pairs) is shown, where +1 is the transcription start site. Only binding site sequences that go across all genomes are shown. Chromosomal locations of the binding site sequences and the full sequences are available in text file format via the 'download result' and 'download FASTA file' links. **(c) **Visualization of elongation factor-2 (E2F)-binding sites in the *CDC2 *upstream region. It is the same region as is shown in Figure 3b, with only the E2F sites highlighted. Other binding sites were suppressed by the toggle.

In order to display the presence of consensus transcription factor binding site sequences on a promoter that spans multiple species, we developed a novel Scalable Vector Graphic (SVG)-based graphical interface to display this information in a promoter-oriented way. Using the PBSS, regulatory regions of genes in multiple species along with the consensus TFD binding site information can be quickly visualized. The interface of PBSS is shown in Figure 3a. PBSS takes as input a set of NCBI Entrez gene IDs or gene names and the selected region to visualize. PBSS automatically retrieves the specified region for each gene in the input set based on the corresponding genome annotation. There are three specific regions that can be searched: the regulatory region of a gene upstream of the transcription start site, upstream from the start codon, and downstream from the stop codon. Alternatively, custom sequences can be specified. Along with the use of TFD, users can also enter arbitrary binding site IUPAC (International Union of Pure and Applied Chemistry) degenerate sequences. If the 'across genomes' option is selected, then only the binding sites that span the selected genomes are reported. In addition to the SVG graphical display, users can also choose to generate tab-delimited text, which can be readily imported into other programs such as Microsoft Excel. A sample SVG graphical output for the gene *CDC2 *(cell division cycle 2) is shown in Figure 3b.

The benefits of the SVG graphical display of the regulatory regions of genes, presented in a regulatory motif-oriented fashion for each species, are numerous (Figure 3b). One major advantage of the SVG graphical display is that it provides dynamic controls such that the user can switch on and off the display for each binding site and change the range of the location. Furthermore, in moving the cursor over individual binding sites, additional information, such as the binding site sequence pattern and the location of the binding site, can be displayed. Interestingly, the *CDC2 *motifs are conserved around the -150 bp region, of which two of the binding sites are elongation factor-2s (E2Fs). In Figure 3c, the same region is displayed with only the E2F-binding sites highlighted. Indeed, this regulatory region has been cloned by Zhu and coworkers [31], and the region was shown to be responsive to E2Fs. Using the GeneACT promoter browser, the arrangement of the binding sites across genomes can be easily visualized. Based on this analysis, the user can identify a potential regulatory region in a faster and more educated fashion than the traditional method of arbitrary sequential deletion analysis. The ease of use and clear presentation should be an attractive feature for experimental biologists.

## Genomic sequence retrieval and Transcription Factor Database search

GeneACT also provides other tools to make promoter analysis easier. The genomic sequence retrieval tool allows the user to retrieve genomic sequences in a FASTA format using relative position with respect to the transcription start site, start codon, or stop codon. When the input has more than one gene name or gene ID, sequences are returned in a concatenated FASTA file. Information about the sequence such as the chromosomal location, gene name, synonyms, and gene ID are printed in the header of the FASTA file. For the genes that are annotated to be on the reverse complement strand, this tool returns the sequence on the reverse complement strand.

**Figure 4:**
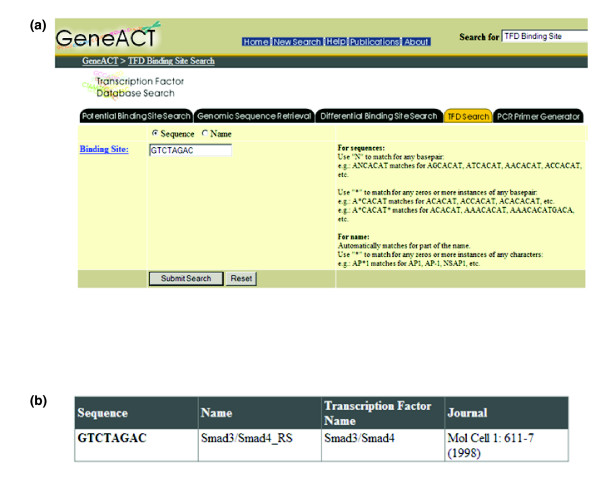
Search transcription factor binding site database. **(a) **Custom transcription factor database based on Transcription Factor Database (TFD). Database can be queried by sequence and name. New entries into the database can be added by the system administrator. **(b) **Display of the search result of a transcription factor binding site. The literature information of the binding site is shown.

TFD search can be used to perform a query in the TFD dataset for binding site sequence or transcription factor name (Figure 4). Other than transcription factor binding sites, miRNA-binding sequences are also important for regulation of gene expression. To keep the database contents up to date, the user can submit putative novel binding site sequences via this tool. All submissions will be curated and deposited into our database. These new binding sites will then be included for the next round of pre-processing for DBSS such that they will be available for searches within all tools in GeneACT. In this way, GeneACT will remain relevant to the current literature. For the most in-depth information on how to use GeneACT, help documentation is available on the website [21].

## Mining gene expression data using differential binding site search

The use of microarrays to elucidate genome-wide gene expression patterns is now standard practice. These microarray experiments generate large sets of differentially expressed genes, but the actual mechanism that controls the differential gene expression cannot readily be deduced using this technique alone. To ascertain the *cis*-regulatory elements that could mediate the differential gene expression patterns, we developed the DBSS tool to explore the distributions of regulatory sequence elements between the differentially expressed genes compared with those of the control genes. A corollary to the importance of *cis*-acting regulatory elements to generating differential gene expression patterns is that some of the co-expressed genes may share a common subset of these elements, and the observed frequency of these elements in the upregulated or downregulated gene set should be greater than in the unchanged gene set.

DBSS tracks the frequencies of *cis*-acting elements conserved in human, mouse, and rat in a given set of genes and reveals the over-represented *cis*-acting elements in comparison with a control gene set. DBSS takes as input two sets of genes: a control set and a regulated set. For the purposes of identifying over-represented transcription factor binding sites in the regulated set, the regulatory regions of each gene in both sets are searched for transcription factor binding sites that are conserved across each genome. At present, we have pre-processed each gene that contains ortholog information in NCBI HomoloGene for the -10,000 bp to +100 bp region centered on the start codon and the -2000 bp to +100 bp region centered on the stop codon for the purposes of looking for enriched transcription factor binding sites. Restricting the binding sites solely to those that span multiple genomes is intended to reduce background noise. However, certain short degenerate binding site sequences may still appear as false positives. Thus, we use the control set of genes to reduce further the false-positive rate because these types of binding sites are also expected to appear with high frequency in this dataset as well.

Specifically, the DBSS calculates the frequency at which each binding site occurs in genes from both the regulated set and control set. The fold change in frequency of each binding site between the regulated and control gene sets is calculated in order to find binding sites that are enriched in the regulated set. For binding sites that do not contribute to the regulation of a particular gene, we expect there to be no relative change in frequency. These genes are then filtered from the results by specifying a lower bound for the 'binding site ratio' option on the search interface. For example, to keep only the binding sites that have three times the frequency in the regulated set versus the control set, one would specify a lower bound of three. By looking at the binding sites that have a large ratio (fold change) between the regulated set genes and control set genes, the binding site sequences that are potentially important to the regulation of a given system under specific conditions or treatments can quickly be determined. In this way, the regulatory mechanism of how the transcription factors regulate a given system can be inferred from the enriched binding site sequences.

## Discovering potential transcription factor participants in a system using differential binding site search

To test whether mining of DNA microarray datasets using DBSS can generate novel insights into the key transcription factors operating in differential gene expression, we downloaded a microarray dataset (GSE1692) deposited in the NCBI Gene Expression Omnibus [32] database by Cam and coworkers [33]. Those investigators investigated cell cycle dependent gene expression in T98G fibrosarcoma cells. They performed gene expression and ChIP-chip analyses of asynchronous cells compared with quiescent cells prepared by removal of serum for 3 days. To analyze the same dataset independently, we first performed *t*-tests for each gene in this dataset and set our threshold at *P *< 0.05 to define genes that were differentially expressed; there were a total of 670 genes in this regulated gene set. We chose the genes that had *P *> 0.7 as our controls; there were a total of 612 genes in this control gene set. The actual *P *values for individual genes are reported in Additional data file 1. Using the DBSS, we analyzed the promoter regions of these genes in the -10,000 bp to +100 bp region relative to the start codon and filtered the results to those binding sites with a threefold change in frequency. As shown in Table 1, E2F-related binding sites dominated the list of search results, suggesting that the E2F family of transcription factors may be involved in the observed difference in gene expression profiles between quiescent and proliferating cells. Indeed, our results were in good agreement with those of Cam and coworkers [33].

To demonstrate independently that some of the genes appearing in our list predicted to contain over-represented E2F binding sites are indeed bound by E2F1 or E2F4 *in vivo*, we conducted a ChIP assay. We used E2F1 and E2F4 antibodies to analyze the occupancies of these two transcription factors on five different promoters in both synchronized and quiescent T98G cells. A brief description of our ChIP methodology is as follows. Approximately 1 × 10^7 ^T98G cells were fixed with formaldehyde (1% final concentration) at room temperature for 10 min. Fixation was stopped by the addition of glycine for 5 min. Cells were washed once with ice-cold phosphate-buffered saline supplemented with protease inhibitors (1 μg/ml phenylmethylsulfonyl fluoride, 1 μg/ml aprotinin, 1 μg/ml pepstatin). Cells were scraped and pelleted in the same buffer. Cell pellets were lysed in 0.5 ml lysis buffer (1% sodium dodecyl sulfate; 10 mmol/l EDTA; 50 mmol/l Tris-HCl [pH 8.0]). Soluble chromatin was prepared by sonication of the cell lysates. Subsequent immunopreciptation and analysis were performed essentially according to the method proposed by Lambert and coworkers [34], except that antibodies against E2F-1 (sc-193; Santa Cruz Biotechnology, Santa Cruz, CA, USA) and E2F-4 (sc-1082; Santa Cruz Biotechnology) were used; 0.1% of total input chromatin was used in the polymerase chain reactions in the input lane.

**Figure 5:**
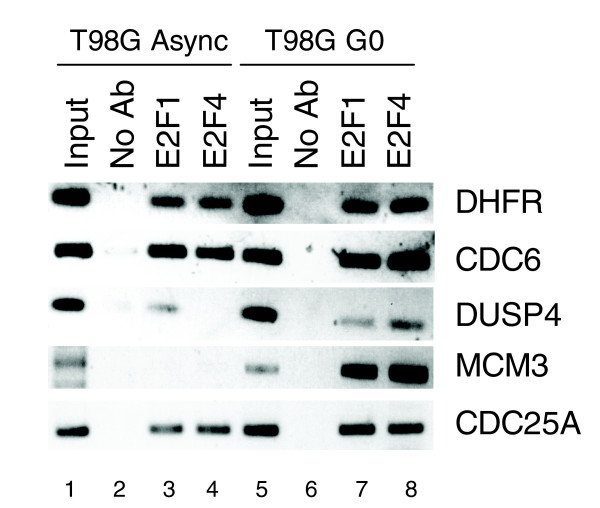
E2F1 and E2F4 occupancies in different promoter regions predicted by differential binding site search. A chromatin immunoprecipitation experiment was performed as described in the text. Mock experiments were done using no antibodies (No Ab), which served as a negative control for the experiment. Input lane represents polymerase chain reactions using 0.1% of total input chromatin. E2F, elongation factor-2.

As shown in Figure 5, all five promoters are indeed targeted by E2F1 or E2F4, although the pattern of binding varies among these five genes. Whereas our ChIP data on *DHFR*, *CDC6*, *CDC25A*, and *MCM3 *are consistent with published results, binding of E2F1 and E2F4 to *DUSP4 *is a novel finding. Thus, based on the results of DBSS, we can gain biological insights similar to those obtained by ChIP-chip analysis.

**Figure 6:**
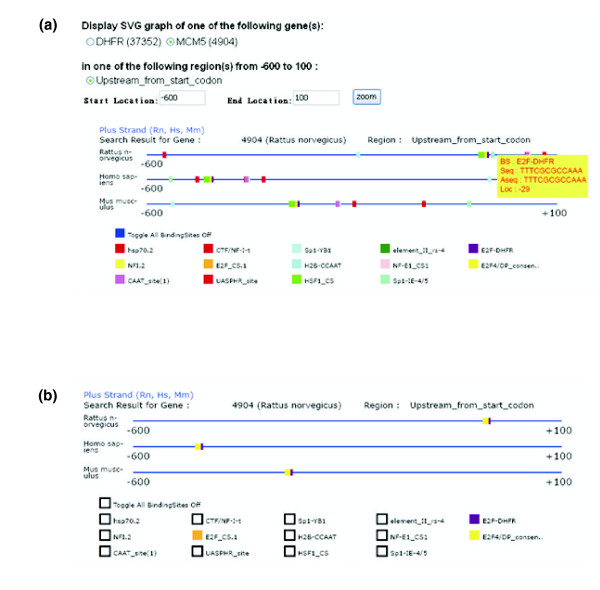
Graphic display of transcription binding sites in the MCM5 promoter region. **(a) **Visualization of the *MCM5 *upstream region. *MCM5 *upstream region (-600 to +100 base pairs) is shown, where +1 is the transcription start site. Only binding site sequences that go across genomes are shown. **(b) **The same region as is shown in panel a, with only the E2F sites highlighted.

**Figure 7:**
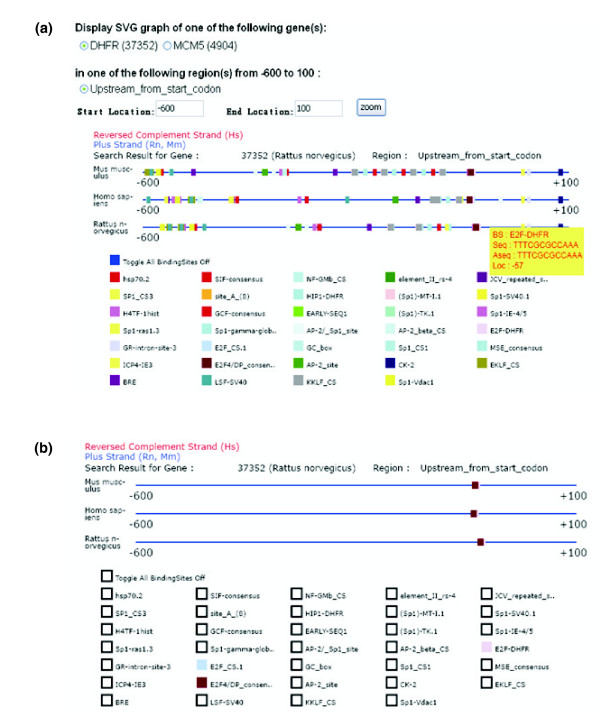
Graphic display of transcription binding sites in the DHFR promoter region. **(a) **Visualization of *DHFR *upstream region. Same parameters were used as for *MCM5 *(see Figure 6). **(b) **Same region as shown in panel a, with only the E2F sites highlighted. E2F, elongation factor-2.

To demonstrate the visualization capabilities of GeneACT, we use the example of serum response factor (SRF), whose binding sites were highly enriched in the regulated gene set. The increased presence of SRF binding sites implies that genes containing this site might be regulated by SRF when cells enter G_1 _from G_0_. Indeed, one of the differentially expressed genes that contributes to the SRF ranking, namely *EGR1*, has been independently shown to be activated by SRF [35]. Genes that contain either E2F or SRF binding sites are listed in Additional data file 3. The location of the putative E2F-binding sites can easily be tracked down using the GeneACT graphical interface of PBSS. The promoter regions (-600 bp to +100 bp) of *MCM5 *(Figure 6a) and *DHFR *(Figure 7a) are shown in the promoter browser using PBSS. Figures 6b and 7b highlight just the E2F binding sites conserved in these promoter regions, respectively. Taken together, our results suggest that DBSS in GeneACT can be a simple but very useful tool to gain novel insights from microarray data quickly.

## Discovering potential microRNA participants in a system using differential binding site search

If the abundance of mRNA is regulated by miRNA, then we would expect that expression levels of miRNAs and their authentic targets should be anti-correlated. Accordingly, computational identification of over-represented miRNA target sites shared among co-regulated genes from DNA microarray data in theory should provide valuable leads to uncover the biologically relevant miRNAs responsible for differential gene expression. To test this hypothesis in a well characterized system, we downloaded and analyzed the dataset created by Lim and coworkers [17]. This investigation was to identify the targets of miR-1 and miR-124 in HeLa cells by overexpression of these two miRNAs independently followed by profiling mRNA transcript levels by DNA microarray analysis. They found that 96 and 174 annotated genes were downregulated by miR-1 and miR-124, respectively. If over-representation of miRNA target sites among co-regulated genes can be exploited to unravel the controlling miRNAs in differential gene expression, then searching the list of 96 or 174 genes using the 3'-UTR search function with the DBSS tool is expected to reveal over-representation of miR-1 or miR-124 target sites, respectively, among these two group of genes. miR-1 and miR-124 are noted for their tissue specificity in mammals. miR-1 is known to be preferentially expressed in heart and skeletal muscle, whereas miR-124 is known to be preferentially expressed in brain [36,37]. Because they are tissue-specific miRNAs, we used each of the datasets as a control for the other.

The results are summarized in Table 2 and Additional data file 4. As predicted, miR-124 target sites ranked among the top of the list in the search result when the regulated gene set input was the miR-124 overexpression experiment. As for miR-1, we found that miR-1 was excluded from our analysis because of the missing orthologous miR-1 mature miRNA sequence in rat, and so it is not discussed further. We note that the target sites for many other miRNAs were also enriched in addition to the miR-124 target sites. This implies that genes that are downregulated by miR-124 also contain miRNA target sites for other miRNAs. It is possible that multiple miRNAs might act on similar sets of genes that are downregulated by miR-124 in the HeLa cell line. Recapturing miR-124 from the DBSS search in GeneACT using the corresponding list of genes determined by DNA microarray analysis suggests that this is a potentially very productive approach to zero in on the miRNAs responsible, at least in part, for a given expression profile.

## Predicting microRNA participants in skeletal muscle differentiation

Myogenic differentiation is a process that leads to the fusion of muscle precursor cells (myoblasts) into multinucleated myofibers in the animal. The C2C12 myoblast cell line serves as a good *in vitro *model for studying skeletal muscle differentiation because these cells are able to differentiate terminally into myotubes when serum is withdrawn from the culture medium [38,39]. To understand the potential involvement of miRNAs in regulating skeletal muscle differentiation and further test our tool, we employed DBSS to analyze a C2C12 differentiation microarray dataset found on NCBI GEO. In this dataset, C2C12 differentiation was studied from day 0 to day 10 of serum withdrawal [40]. Our control genes were those that were upregulated at all time points compared with the control undifferentiated myoblasts. We hypothesized that these genes are less likely to be changed by the miRNA because they are upregulated in the time course and the nature of miRNA regulation is to downregulate the expression of mRNA. To perform the analysis, we compared the cells at day 2 of differentiation with those at day 0 (Additional data files 5 and 6).

The result is summarized in Table 3. Our *in silico *analysis of the C2C12 microarray gene expression profile using DBSS implied that at least 14 miRNA target sites are over-represented in downregulated mRNAs during myogenic differentiation in C2C12 cells, suggesting that some of these microRNAs may be differentially expressed during myogenic differentiation and contribute to the mRNA expression profile. Recently, Chen and colleagues [16] investigated a number of miRNA expression profiles during C2C12 differentiation using a miRNA microarray. Their miRNA array expression data revealed that miR-133a, miR-206, and miR-130a were ranked at the top of the list of a few miRNAs that were upregulated upon myogenic differentiation. In comparing our *in silico *predictions with their experimental results, we found that our analysis recaptured miR-133a, miR-206, and miR-130a target sites as the most enriched in differentially expressed genes. Therefore, a differential miRNA target site search can generate predictions consistent with experimental results in this system.

It has previously been demonstrated *in vitro *that more than two miRNA target sites in a given 3'-UTR seem to boost the efficacy of miRNA-mediated gene repression [41]. To test whether implementing the more stringent requirement that at least two or three conserved sites are present on any one mRNA will improve the accuracy of predicting the miRNA participants in the skeletal muscle differentiation dataset, we compared the output of the more than two target site prediction with the result of the microRNA microarray experiment. As shown in Table 3, introduction of this additional constraint did not improve the performance of the prediction when compared with the experimental results. Therefore, it remains to be determined whether multiplicity of miRNA target sites in mRNA can be used as a reliable criterion for predicting the authenticity of miRNA targets.

## Discussion

GeneACT was developed to display and analyze regulatory regions across human, mouse and rat genomes, and it enables identification of putative *cis*-acting elements that are evolutionarily conserved across species for all orthologous genes. A comparative, online, web-based, graphically oriented promoter browser was developed for the public domain. Using the DBSS, insights can be gained into a particular system in which transcription factors might be involved. GeneACT enables integration of *cis*-regulatory sequences identified by a comparative genomics approach with microarray expression profiling data to explore the underlying gene expression regulatory networks.

To illustrate the uniqueness of GeneACT, we compared GeneACT with different existing software. The comparison is summarized in Table 4. There are three distinct features that separate GeneACT from other related programs, the first of which is that GeneACT is the only open source online software that allows identification of over-represented miRNA target sites from a list of genes of interest.

Second, GeneACT employs the TFD database and pattern matching for *in silico *annotation or prediction of potential transcription factor binding sites. Virtually all other programs make use of the position weight matrix (PWM)-based TRANSFAC [11] and related JASPAR databases [42]. Because transcription factors tend to bind short and degenerate sequences, the PWM-based approach provides better definition of transcription factor binding properties based on binding affinity. This method has proved to be very effective for *in silico *prediction of prokaryotic transcription factor binding sites [43,44]. However, there are significant limitations for a PWM-based approach for analysis of mammalian transcription factor binding sites [45,46]. A PWM assumes that the recognition sequence is of fixed length and each base contributes independently to the total binding energy of the transcription factor/DNA complex. In mammalian systems, binding affinity may not be a reliable predictor for biologically relevant binding sites [46]. One of the major features of transcriptional regulation in eukaryotic systems is combinatorial control featuring two or more transcription factors binding synergistically to their target sites [47,48]. The context of the binding site is often more important than individual binding sites. We chose to use the TFD database because almost all of the transcription factor binding sites documented in the database were defined experimentally (for example, by reporter assays). The TFD contains more than 7000 characterized binding sites from a variety of biologic contexts. These binding sites are naturally selected for function during evolution. Thus, using TFD in our *in silico *analysis provides an alternative and perhaps more relevant approach to identification of putative transcription factor binding sites in the flanking regions of genes of interest. Given the findings that no single transcription factor binding site discovery program is superior from a number of comparative studies and that using multiple independent programs improves the performance of prediction [49], GeneACT is a valuable addition to existing tools.

The third and final distinct feature that separates GeneACT from other related programs is that the output of GeneACT is geared toward easy visualization and pattern recognition. It is designed to be a simple, freely available tool for experimental biologists to navigate promoter regions and discover the significance of a given DNA sequence based on comparative genomic analysis and DNA microarray data. Extensive tutorials and help documents are available on our website help page to guide users through different tools on this site. A major feature of GeneACT is the miRNA target site search capability. This is crucial, given that up to one-third of human genes could be targeted for regulation by miRNA [50], in addition to regulation by transcription factors. It is therefore important to investigate both transcription factors and miRNAs when searching for critical genes that may be responsible for differential gene expression. By integrating both transcription factor binding sites and miRNA target sites into DBSS, we provide a more comprehensive analysis of DNA microarray datasets. Indeed, we showed that GeneACT accurately predicted the involvement of E2F during cell cycle progression and involvement of certain miRNAs during muscle cell differentiation from DNA microarray datasets.

The quality of predictions of critical *cis*-regulatory elements involved in differential gene expression depends heavily on the reliability of transcription factor recognition and miRNA target site prediction. Accurate computational prediction of miRNA target sites is still a very challenging task because of insufficient experimental data [51]. For example, it is not clear whether the length of the 3'-UTR where the putative miRNA target sites reside contributes to the efficacy of gene repression. A definitive answer to this question is likely to dictate how to factor the length of the 3'-UTR into reliable prediction scores.

GeneACT is open source online software and is relative easy to upgrade. We expect DBSS will improve significantly as miRNA target site prediction and transcription factor binding site recognition becomes more reliable. Moreover, in the future we plan to add additional genomes to GeneACT as they become available. Even so, it is possible for researchers interested in other species to use GeneACT by taking advantage of the input sequence feature and/or input binding site feature of PBSS. In this way, we expect researchers from different and diverse fields to find a valuable resource in GeneACT.

## Additional data files

The following additional data are available with the online version of this paper. Additional data file 1 is a table containing the original DNA microarray data generated by Cam and coworkers [33] used for DBSS; the gene IDs of regulated and control gene sets used for the search are listed. Additional data file 2 is a table containing the full list that is summarized in Table 1. Additional data file 3 is a table containing cell cycle regulated genes containing E2F or SRF binding sites. Additional data file 4 is a table containing the full list that is summarized in Table 2; the gene IDs of the miR-124 dataset used for the search are listed. Additional data file 5 is a table containing the original DNA microarray data generated by Tomczak and coworkers [40] used for the DBSS; gene IDs of regulated and control gene sets used for the search are listed. Additional data file 6 is a table containing the full lists summarized in Table 3.

## Supplementary Material

Additional data file 1 Table containing the original DNA microarray data generated by Cam and coworkers [33] used for DBSS; the gene IDs of regulated and control gene sets used for the search are listed. Click here for file

Additional data file 2 Table containing the full list that is summarized in Table 1. Click here for file

Additional data file 3 Table containing cell cycle regulated genes containing E2F or SRF binding sites. Click here for file

Additional data file 4 Table containing the full list that is summarized in Table 2; the gene IDs of the miR-124 dataset used for the search are listed. Click here for file

Additional data file 5 Table containing the original DNA microarray data generated by Tomczak and coworkers [40] used for the DBSS; gene IDs of regulated and control gene sets used for the search are listed. Click here for file

Additional data file 6 Table containing the full lists summarized in Table 3. Click here for file

## Figures and Tables

**Table 1:** Binding site sequences that are enriched in quiescent T98G cells versus asynchronous T98G cells from DBSS

Name of binding site	Transcription factor	Sequence	Ratio	Regulated gene frequency	Control gene frequency
^a^E2F4/DP_consensus	E2F4/DP	TTTSGCGCS	8.221	9	1
element_II_rs-4	element_II_rs-4	TTTCGCG	7.307	8	1
^a^E2F_CS	E2F	TTTTSSCGS	7.307	8	1
AP-1-erk1	AP-1	CAGACTAA	6.394	7	1
m4-AP-1_site	AP-1	GTGAGTAA	5.481	6	1
E1A-BS4	E1A-BS4	GTCAAAGT	5.481	6	1
^a^E2F_site_(2)	E2F	TTTGGCGC	5.481	6	1
E1A-BS5	E1A-BS5	TCTCAGGTG	5.481	6	1
epsilon-NRA-FP2	undefined	GAGATACC	5.481	6	1
HC5	HC5	CCGAAAC	4.567	10	2
TB3	NF-IL-6	AACTGGAAA	4.567	5	1
GCN4_CS1	GCN4	ATGASTCAT	4.567	10	2
MyoD-MLC_(1)	MyoD	CCAGCTGGC	4.567	5	1
Sp1-t-PA	Sp1	ACCCCGCCC	4.567	10	2
CArG_CS	SRF	CCAWATWWGG	4.567	5	1
RC1/RC2-CYC	RC1/RC2	TGACCGA	4.567	5	1
DHFR-undefined-site-1	DHFR-undefined-site-1	GGATTGGC	4.110	9	2
TopoII_RS	Topoisomerase II	RNYNNCNNGYNGKTNYCY	4.110	9	2
element_II-rs-1	element_II-rs-1	GGCGTAA	3.654	4	1
C/EBP-TTRS3	C/EBP	TCTTACTC	3.654	8	2
Sp1-Vdac2	Sp1	CCTCGCCTC	3.654	4	1
glide/gcm_CS	glide/gcm	ATRCGGGY	3.654	4	1
spB-4bp	STAT3	TTCCGGAA	3.654	4	1
C/EBP-AT-Site-C.2	C/EBP	TCTTAAGC	3.654	8	2
PUT2_UAS2; PUT2_UAS.2	PUT3	GAAGCCGA	3.654	4	1
NFkB_CS2	NF-kB	RGGGRMTYYCC	3.349	11	3
^a^E2F_site_(3)	E2F	TTGGCGC	3.288	18	5
NF-E2_CS	NF-E2_CS	TGACTCAGC	3.197	7	2
^a^E2F_CS.2	E2F	SCGSGAAAA	N/A	7	0
AluA	AluA	GGAGGCTGAGGCA	N/A	6	0
^a^E2F_CS.1	E2F	TTTCGCGC	N/A	5	0
Swi4-mdscan-motif-3	Swi6	AAACGCG	N/A	5	0
E-box/ATF/CREB_site	Ebox protein/ATF/CREB	GTGACGCA	N/A	5	0
GCN4-his3-189	GCN4	ATGACTCAT	N/A	4	0
GCF-beta-actin_(2)	GCF	GCGCGGGCCG	N/A	4	0
Sp1-XIST_(1)	Sp1	GGCCACGCC	N/A	4	0
rMT-III-motif-9	undefined	CAGGCACCT	N/A	4	0
DBP-CS	DBP	RTTAYGTAAR	N/A	4	0
CDF1_RS	CDF1	CTAAATAC	N/A	4	0
alphaA-crystallin-PE2A	AP-1	CTGACTCAC	N/A	4	0
^a^E2F-myc	E2F	GCGGGAAAA	N/A	4	0

A selected list is shown here; see Additional data file 1 for the full list. Only binding site sequences with a fold change in frequency of occurrence of greater than three are shown. ^a^E2F-binding sites are highlighted in grey. Ratio of 'N/A' denotes binding site sequences that can only be found in either the control or regulated gene set. DBSS, differential binding site search; E2F, elongation factor-2.

**Table 2:** Summary of the search results for the miRNA target sites enriched in the HeLa cells transfected with miR-124 vs. miR-1

miRNA name	Sequence	Ratio	Number of miR-124 target sites	Number of miR-1 target sites (control)
miR-185	UGGAGAGAAAGGCAGUUC	3.5409836	6	1
^a^miR-124a	UUAAGGCACGCGGUGAAUGCCA	3.5409836	12	2
miR-145	GUCCAGUUUUCCCAGGAAUCCCUU	3.5409836	6	1
miR-22	AAGCUGCCAGUUGAAGAACUGU	-1	8	0
miR-337	UCCAGCUCCUAUAUGAUGCCUUU	-1	7	0
miR-150	UCUCCCAACCCUUGUACCAGUG	-1	6	0
miR-141	UAACACUGUCUGGUAAAGAUGG	-1	6	0
miR-19b	UGUGCAAAUCCAUGCAAAACUGA	-1	4	0
miR-200a	UAACACUGUCUGGUAACGAUGU	-1	3	0
miR-9	UCUUUGGUUAUCUAGCUGUAUGA	-1	3	0
miR-31	GGCAAGAUGCUGGCAUAGCUG	-1	3	0

Shown here is a selected list of the most enriched miRNA target sites when HeLa cells are transfected with miR-124; see Additional data file 4 for the full list. ^a^miR-124 target sites are highlighted. DBSS, differential binding site search; miRNA, microRNA.

**Table 3:** Summary of the results for the miRNA target sites that are enriched in C2C12 myogenic differentiation (day 0 versus day 2) from DBSS

miRNA name	Sequence	Ratio	Regulated frequency	Control frequency
One conserved site				
miR-206	UGGAAUGUAAGGAAGUGUGUGG	4.075358	26	1
miR-7	UGGAAGACUAGUGAUUUUGUUG	3.1348907	20	1
miR-301	CAGUGCAAUAGUAUUGUCAAAGC	3.1348907	20	1
miR-23a	AUCACAUUGCCAGGGAUUUCC	2.8214017	18	1
miR-138	AGCUGGUGUUGUGAAUC	2.8214017	18	1
miR-211	UUCCCUUUGUCAUCCUUCGCCU	2.2727958	58	4
miR-29c	UAGCACCAUUUGAAAUCGGU	2.037679	13	1
miR-133a	UUGGUCCCCUUCAACCAGCUGU	1.9788998	101	8
miR-194	UGUAACAGCAACUCCAUGUGGA	1.9593067	25	2
miR-30c	UGUAAACAUCCUACACUCUCAGC	1.8809344	12	1
miR-130a	CAGUGCAAUGUUAAAAGGGCAU	-1	18	0
miR-216	UAAUCUCAGCUGGCAACUGUG	-1	12	0
miR-196b	UAGGUAGUUUCCUGUUGUUGG	-1	10	0
miR-140	AGUGGUUUUACCCUAUGGUAG	-1	10	0

Two conserved sites				
miR-324-5p	CGCAUCCCCUAGGGCAUUGGUGU	2.5079126	32	2
miR-193	AACUGGCCUACAAAGUCCCAG	2.037679	13	1
miR-150	UCUCCCAACCCUUGUACCAGUG	2.037679	13	1
miR-125b	UCCCUGAGACCCUAACUUGUGA	-1	18	0
miR-133a	UUGGUCCCCUUCAACCAGCUGU	-1	17	0
miR-152	UCAGUGCAUGACAGAACUUGGG	-1	11	0
miR-204	UUCCCUUUGUCAUCCUAUGCCU	-1	9	0
miR-17	CAAAGUGCUUACAGUGCAGGUAGU	-1	8	0
miR-211	UUCCCUUUGUCAUCCUUCGCCU	-1	8	0

Three conserved sites				
miR-296	AGGGCCCCCCCUCAAUCCUGU	2.037679	26	2
miR-34a	UGGCAGUGUCUUAGCUGGUUGUU	-1	11	0
miR-125a	UCCCUGAGACCCUUUAACCUGUG	-1	7	0
miR-330	GCAAAGCACACGGCCUGCAGAGA	-1	7	0
miR-345	UGCUGACUCCUAGUCCAGGGC	-1	6	0
miR-320	AAAAGCUGGGUUGAGAGGGCGAA	-1	6	0
miR-125b	UCCCUGAGACCCUAACUUGUGA	-1	5	0
miR-449	UGGCAGUGUAUUGUUAGCUGGU	-1	5	0
miR-150	UCUCCCAACCCUUGUACCAGUG	-1	5	0
miR-133a	UUGGUCCCCUUCAACCAGCUGU	-1	5	0
miR-199a	CCCAGUGUUCAGACUACCUGUUC	-1	4	0
miR-193	AACUGGCCUACAAAGUCCCAG	-1	4	0
miR-205	UCCUUCAUUCCACCGGAGUCUG	-1	4	0

Selected lists are shown here; see Additional data file 6 for the full lists. Findings are subdivided into results for one conserved site, results for two conserved sites, and results for three conserved sites. In all cases, miR-133a remained as one of the top hits. If a given miRNA target site is absent in the control gene list, then we listed the ratio as 'N/A', which denotes the unique presence of this particular target site in the regulated gene list. DBSS, differential binding site search; miRNA, microRNA.

**Table 4:** Summary of the comparison between different similar tools in the public domain to that of GeneACT

	GeneACT [30]	oPOSSUM [13]	OTFBS [52]	Clover [53]	Whole Genome rVISTA beta [3]	CRÈME [15]
Graphical user interface	Web based	Web based	Web based	Command line tool for Linux, UNIX and Mac OS X	Web based	Web based
Genomic display	Yes	No	No	No	Yes	No
Type	Promoter specific	N/A	N/A	N/A	Gene specific	N/A
Display motifs on custom sequences	Yes	N/A	N/A	N/A	N/A	N/A
Allow custom sequences input	Yes	N/A	N/A	N/A	N/A	N/A
Source of the transcription factor binding sites	TFD	JASPAR	TRANSFAC	JASPAR	TRANSFAC	TRANSFAC
Number of binding sites in the database	>7000	111	>500	111	>500	>500
Search for over-represented binding site	Yes	Yes	Yes	Yes	Yes	Yes
In promoter region	Yes	Yes	Yes	Yes	Yes	Yes
In 3'-UTR	Yes	No	No	No	No	No
Across genomes	Human, mouse and rat	Human and mouse	No	No	Yes	Human
Species supported (using Locuslink, Entrez gene ID, Ensembl ID, etc.)	Human, mouse and rat	Human and mouse	No	No	Mouse only	Human
Number of genes/sequences allowed	Unlimited IDs	Unlimited IDs	200 sequences	Not tested	Unlimited IDs	Unlimited IDs
Allow custom control gene set	Yes	Yes	No	Yes	No	No
Correctly predicted E2F4 binding sites in the T98G dataset	Yes	No	N/A	N/A	N/A	Yes
Identified E2F-binding sites in the T98G dataset	Yes	Yes	N/A	N/A	N/A	Yes
Search for potential miRNA involved in the dataset	Yes	No	No	No	No	No
Source for the miRNA seed sequences	miRbase	N/A	N/A	N/A	N/A	N/A
Across genomes	Human, mouse and rat	N/A	N/A	N/A	N/A	N/A
Species supported	Human, mouse and rat	N/A	N/A	N/A	N/A	N/A
Number of genes/sequences allowed	Unlimited	N/A	N/A	N/A	N/A	N/A
Allow custom control gene set	Yes	N/A	N/A	N/A	N/A	N/A

Only programs that have web interfaces and allow unlimited Gene IDs are tested using the T98G dataset. The website addresses for each of the programs evaluated are given in the references provided in the top row. N/A represents a category that is not available. E2F, elongation factor 2; miRNA, microRNA; UTR, untranslated region.
